# Dysphasia is associated with diffusion-weighted MRI abnormalities in patients with transient neurological symptoms

**DOI:** 10.1007/s10072-020-04258-z

**Published:** 2020-02-07

**Authors:** Zejin Jia, Yangguang Song, Wenli Hu

**Affiliations:** 1 Department of Neurology, Beijing Chaoyang Hospital, Capital Medical University, Beijing, 100020 China; 2 Department of Pathology, Beijing Chaoyang Hospital, Capital Medical University, Beijing, 100020 China

**Keywords:** Transient neurological symptoms, Diffusion-weighted imaging, Acute ischemic lesions, Dawson score, The Diagnosis of TIA score

## Abstract

**Background:**

The clinical characteristics of diffusion-weighted imaging (DWI) abnormalities after transient neurological symptoms are of great significance for the early diagnosis and urgent intervention of transient ischemic attack (TIA). This study was aimed to investigate the clinical characteristics associated with acute DWI lesions in transient neurological symptoms.

**Methods:**

We retrospectively recruited 302 patients with transient neurological symptoms. According to DWI findings, they were divided into DWI positive and DWI negative group. The clinical characteristics and the TIA-related scores such as ABCD2, ABCD3, ABCD3I, Dawson score, and the Diagnosis of TIA (DOT) score were compared between the two groups. Logistic regression analysis and receiver operating characteristic curves were used to identify the independent factors and compare the predictive value of different TIA scores for acute DWI lesions.

**Results:**

A total of 302 patients were enrolled in this study. The mean age was 61.8 years, and 67.2% were male. We found 89 (29.5%) patients with DWI positivity. Logistic regression analysis showed the characteristic associated with DWI lesions was dysphasia (OR 2.226, 95% CI 1.220–4.062). The area under the curve for Dawson score and the DOT score was 0.610 (95% CI 0.543–0.678) and 0.625 (95% CI 0.559–0.691), respectively.

**Conclusion:**

We found that DWI lesions were detected in 29.5% of patients with transient neurological symptoms and were associated with dysphasia. Dawson score and DOT score could have a higher predictability of DWI lesions in patients with transient neurological symptoms.

## Introduction

A new tissue-based definition of transient ischemic attack (TIA) is a transient episode of focal neurological deficits consistent with cerebrovascular accident lasting less than 24 h and with the absence of a diffusion-weighted imaging (DWI) lesion detected by MRI [1–4]. TIA is treated as a medical emergency and may precede ischemic stroke that causes permanent neurological deficits. The stroke risk after TIA may be as high as 10% in the first week [5]. Identifying and treating patients with TIA is an effective way to prevent stroke [6]. DWI is a mandatory tool in the diagnosis of a TIA [7]. However, in many emergency settings, patients with transient neurological symptoms are difficult to have urgent brain MRI due to cost issues [8]. Several studies have shown that diffusion-weighted imaging (DWI) lesions were associated with a high risk of recurrent ischemic stroke after transient neurological symptoms [9–11]. The frequency of positive DWI findings in patients with transient neurological symptoms varied from 9 to 67% in some prior studies [12]. The clinical features associated with DWI lesions after transient neurological symptoms are of great significance for the early diagnosis and urgent intervention of TIA [13]. It has been reported that the presence of acute DWI lesions in transient neurological symptoms were associated with motor weakness, aphasia, dysarthria, left hemispheric presenting symptoms, National Institutes of Health Stroke Scale (NIHSS) score of ≥ 10 at admission, time from onset to DWI longer than 24 h, intracranial large artery atherosclerosis, and old brain infarctions on MRI [14–17]. However, as for the incidence of positive DWI in Chinese patients with transient neurological symptoms and its’ independent predictors for this positive DWI are poorly understood.

Recently, the Diagnosis of TIA (DOT) score has been proposed [18]. It is a new and internally validated web and mobile app-based diagnostic tool which encompasses both brain and retinal TIA. However, as a promising diagnostic tool for TIA, DOT score still needs independent external validation before it can be widely utilized.

Thus, our study was aimed to investigate the clinical characteristics associated with the presence of acute DWI lesions in Chinese patients with transient neurological symptoms and tested the validation of DOT score in China.

## Methods

### Patients

We retrospectively identified patients with transient neurological symptoms lasting less than 24 h, who were admitted to the neurology department of Beijing Chaoyang Hospital, Capital Medical University from January 2016 to February 2019. All the patients underwent DWI within 5 days after admission. We used a 3.0-T MRI to evaluate whether acute ischemic lesions were present on admission. Sequences included diffusion-weighted, T1-weighted, and T2-weighted imaging; fluid-attenuated inversion recovery (FLAIR); and magnetic resonance angiography (MRA). Acute DWI lesions were defined by the areas of high signal intensity on DWI with restricted diffusion. All MRI scans were read by experienced neuroradiologists. Based on the results of DWI, patients were divided into two groups: DWI-positive and DWI-negative group.

We collected a broad range of clinical, laboratory, and radiological data from all patients: the baseline characterizations such as age and gender, clinical symptoms, duration of symptoms, time from onset to MRI, vascular risk factors including hypertension, diabetes mellitus, atrial fibrillation, hyperlipidemia, smoking and alcohol drinking, history of ischemic stroke and coronary artery disease, ABCD2 [19], ABCD3, ABCD3I [20], Dawson score [21], DOT score [18] at admission, the laboratory blood tests on admission recorded for platelet count(PLT), albumin(ALB), prealbumin(PAB), cholesterol(CHOL), low-density lipoprotein(LDL), triglyceride(TG), blood urea nitrogen(BUN), creatinine(Cr), uremic acid(URIC), calcium(Ca), phosphonium(P), fibrinogen(Fbg), fasting blood-glucose(FBG), degree of cerebral artery stenosis in MRA and computed tomographic angiography(CTA), the intimal medial thickness(IMT) of carotid artery in carotid duplex ultrasonography, and treatment after admission. The degree of carotid and intracranial arterial stenosis was classified as over 25%, 50%, and 75% narrowing and occlusion.

The study was approved by the ethics committee of Beijing Chaoyang Hospital and performed in accordance with the Declaration of Helsinki. All subjects provided written informed consent.

### Statistics analysis

Statistical analyses were performed using IBM SPSS Statistics 21. All statistics were presented as mean ± standard deviation (SD) for continuous variables with normal distribution, the median and interquartile range for continuous variables with non-normal distribution, and counts and proportions for categorical variables. We performed *t* test, chi-square test, and the nonparametric Mann–Whitney U test to compare the characteristics between the two groups. A *P* value of less than 0.05 was considered significant. Logistic regression analysis was applied to identify independent predictors of acute DWI lesions in patients with transient neurological symptoms. The results were presented as estimates of relative risk by odds ratio (OR) with a 95% confidence interval (CI). Receiver operating characteristic curve (ROC) analysis was used to compare the predictive values of various scores with regard to acute DWI lesions in patients with transient neurological symptoms.

## Results

We retrospectively recruited 302 patients with transient neurological symptoms who underwent DWI within 5 days after admission. DWI lesions were detected in 89 (29.5%) of the 302 patients. Table 1 shows the clinical characteristics of DWI-positive and DWI-negative group at baseline. The results of blood pressure on admission, the laboratory blood tests, the other examination, and the scores of TIA with the two groups are presented in Table 2.

**Table 1:** The baseline clinical characteristics of patients in DWI-positive and DWI-negative group

Variables	DWI-positive (*n* = 89)	DWI-negative (*n* = 213)	*P* value
Age, years	62 (53,69)	63 (55.5,70.5)	0.430
Male, *n* (%)	71 (79.8%)	132 (62%)	0.003^a^
Risk factors, *n* (%)			
Hypertension	57 (64%)	136 (63.8%)	1.000
Diabetes mellitus	30 (33.7%)	61 (28.6%)	0.410
Coronary artery disease	6 (6.7%)	24 (11.3%)	0.294
Hyperlipidemia	72 (80.9%)	146 (68.5%)	0.034^a^
History of stroke	16 (18.0%)	43 (20.2%)	0.751
Atrial fibrillation	2 (2.2%)	7 (3.3%)	1.000
Smoking	56 (62.9%)	91 (42.7%)	0.002^a^
Alcohol drinking	35 (39.3%)	49 (23%)	0.005^a^
Clinical features, n (%)			
Motor weakness	62 (69.7%)	119 (55.9%)	0.029^a^
Dysphasia	43 (48.3%)	63 (29.6%)	0.002^a^
Sensory disturbance	29 (32.6%)	72 (33.8%)	0.894
Dizziness	29 (32.6%)	67 (31.5%)	0.892
Ataxia	2 (2.2%)	13 (6.1%)	0.245
Amnesia	4 (4.5%)	4 (1.9%)	0.241
Loss of consciousness	5 (5.6%)	19 (8.9%)	0.484
Diplopia	5 (5.6%)	10 (4.7%)	0.774
Homonymous hemianopia	3 (3.4%)	5 (2.3%)	0.697
Time from TIA to MRI, days	4.5 (3,9.25)	6 (3,11)	0.161
Symptom duration, *n* (%)			0.238
< 10 min	33 (37.1%)	99 (47.4%)	
10–59 min	39 (43.8%)	73 (34.9%)	
> 60 min	17 (19.1%)	37 (17.7%)	
Treatment, *n* (%)			0.000^a^
Aspirin	24 (27%)	80 (44.9%)	
Clopidogrel	10 (11.2%)	51 (28.7%)	
Dual antiplatelet	54 (60.1%)	43 (24.2%)	
Anticoagulation	1 (1.1%)	4 (2.2%)	

Data shown: median (interquartile range) or counts (%)

DWI, diffusion-weighted imaging

^a^*P* value less than 0.05

**Table 2:** Results of blood pressure, laboratory tests, examinations, and the TIA-related scores

Variables	DWI-positive (*n* = 89)	DWI-negative (*n* = 213)	*P* value
Systolic pressure, mmHg	151.78 ± 19.119	145.06 ± 20.457	0.008^a^
Diastolic pressure, mmHg	86 (78.5, 92.5)	80 (73, 89.5)	0.002^a^
PLT, *10^9/L	200.60 ± 50.27	216.29 ± 47.65	0.012^a^
ALB, g/L	42.3 (39.95, 44.25)	41.9 (39.125, 44.5)	0.679
PAB, g/L	0.25 (0.21, 0.295)	0.25 (0.2, 0.29)	0.426
CHOL, mmol/L	4.57 (3.78, 5.095)	4.455 (3.848, 5.36)	0.569
LDL, mmol/L	2.8 (1.95, 3.2)	2.6 (2, 3.275)	0.873
TG, mmol/L	1.66 (1.145, 2.365)	1.46 (1.06, 2.225)	0.206
BUN, mmol/L	5.11 (4.34, 5.92)	4.93 (4.405, 5.952)	0.908
Cr, umol/L	67.7 (59.8, 80)	66.5 (55.775, 74.875)	0.127
URIC, umol/L	330.73 ± 88.602	338.53 ± 86.554	0.479
Ca, mmol/L	2.253 ± 0.132	2.266 ± 0.134	0.474
P, mmol/L	1.064 ± 0.242	1.108 ± 0.220	0.121
Fbg, mg/dl	256.3 (229.5, 309.4)	270.3 (228.9, 306.2)	0.428
FBG, mmol/L	6.27 (5.045, 8.565)	5.55 (4.7, 7.055)	0.006^a^
Carotid stenosis, *n* (%)			0.179
No stenosis	55 (64.0%)	153 (74.3%)	
Mild stenosis	15 (17.4%)	28 (13.6%)	
Moderate stenosis	6 (7.0%)	13 (6.3%)	
Severe stenosis	9 (10.5%)	8 (3.9%)	
Occlusion	1 (1.2%)	4 (1.9%)	
Intracranial arterial stenosis, *n* (%)			0.016^a^
No stenosis	30 (34.9%)	117 (56.8%)	
Mild stenosis	20 (23.3%)	33 (16.0%)	
Moderate stenosis	10 (11.6%)	19 (9.2%)	
Severe stenosis	11 (12.8%)	17 (8.3%)	
Occlusion	15 (17.4%)	20 (9.7%)	
IMT, cm	0.28 (0.240, 0.368)	0.255 (0.180, 0.370)	0.038^a^
ABCD2 score	4 (3, 5)	4 (3, 5)	0.018^a^
ABCD3 score	5 (4, 6)	5 (3, 6)	0.002^a^
ABCD3I score	8 (6, 9)	5 (3, 6)	0.000^a^
Dawson score	7.55 ± 1.29	7.04 ± 1.43	0.004^a^
DOT score	1.341 (0.352, 3.717)	0.842 (− 1.407, 2.185)	0.001^a^

Data shown: mean ± standard deviation, median (interquartile range) or counts (%)

*DWI*, diffusion-weighted imaging; *PLT*, platelet count; *ALB*, albumin; *PAB*, prealbumin; *CHOL*, cholesterol; *LDL*, low density lipoprotein; *TG*, triglyceride; *BUN*, blood urea nitrogen; *Cr*, creatinine; *URIC*, uremic acid; *Ca*, calcium; *P*, phosphonium; *Fbg*, fibrinogen; *FBG*, fasting blood-glucose; *IMT*, the intimal medial thickness; *DOT score*, the Diagnosis of TIA score

^a^*P* value less than 0.05

There were significant differences in gender, dysphasia, motor weakness, hyperlipidemia, smoking, alcohol drinking, systolic pressure, diastolic pressure, platelet count, FBG, and the severity of intracranial stenosis between the two groups. Besides, the DWI-positive group had a higher level of IMT of the carotid artery. Logistic regression analysis showed that acute DWI lesions were independently correlated with dysphasia (OR 2.226, 95% CI 1.220–4.062) (Table 3).

**Table 3:** Clinical symptoms associated with DWI lesions in logistic regression analysis

Factors	OR	95% CI	*P* value
Dysphasia	2.226	1.220, 4.062	0.009^a^
Male	1.494	0.634, 3.521	0.358
Hypertension	0.758	0.399, 1.441	0.398
Diabetes mellitus	1.066	0.505, 2.250	0.867
Coronary artery disease	0.393	0.120, 1.291	0.124
Hyperlipidemia	1.609	0.805, 3.215	0.178
History of stroke	0.815	0.378, 1.760	0.603
Atrial fibrillation	0.472	0.044, 5.067	0.536
Smoking	1.294	0.604, 2.772	0.508
Alcohol drinking	1.155	0.566, 2.355	0.692
Systolic pressure	1.012	0.993, 1.032	0.209
Diastolic pressure	1.015	0.985, 1.045	0.345
PLT	0.994	0.987, 1.000	0.051
LDL	0.825	0.592, 1.150	0.256
Fbg	0.999	0.995, 1.003	0.543
FBG	1.069	0.963, 1.186	0.211
Carotid stenosis	1.137	0.829, 1.560	0.426
Intracranial stenosis	1.222	0.990, 1.508	0.062

*DWI*, diffusion-weighted imaging; *PLT*, platelet count; *LDL*, low density lipoprotein; *Fbg*, fibrinogen; *FBG*, fasting blood-glucose

^a^*P* value less than 0.05

**Fig. 1:**
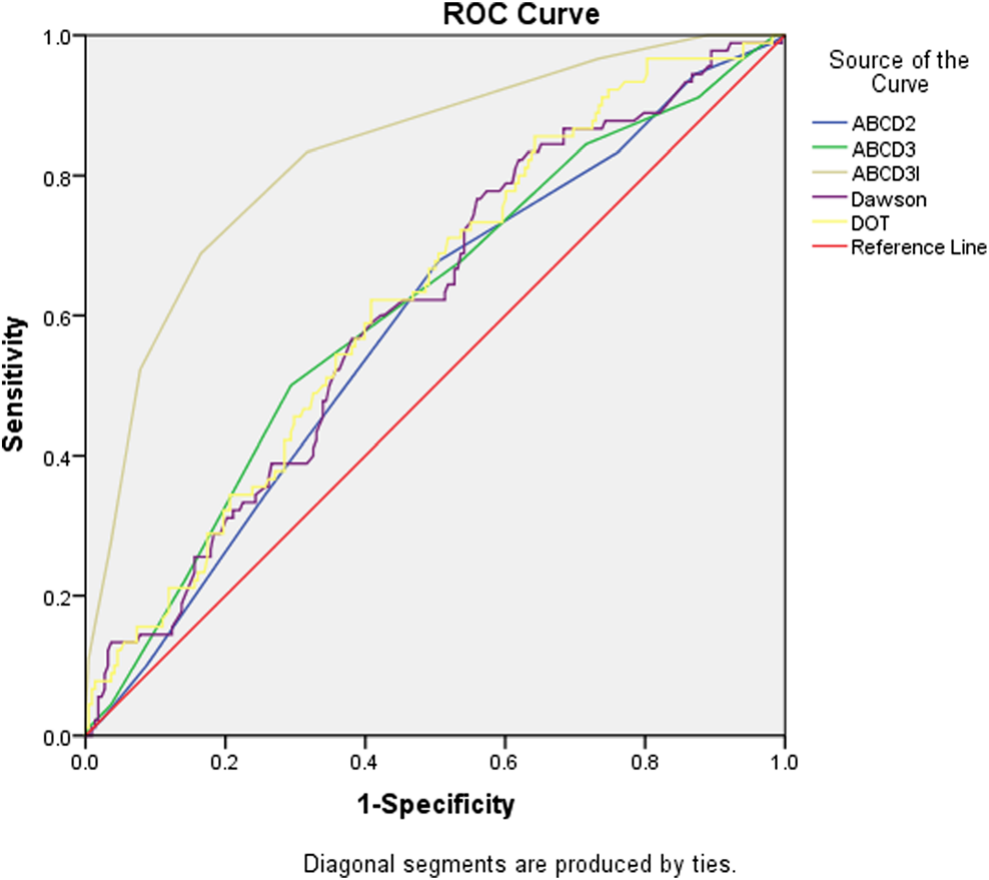
Receiver operating characteristic curve analysis for various scores. DOT, the Diagnosis of TIA score.

In addition, the ABCD2, ABCD3, ABCD3I, Dawson score, and DOT score were significantly higher in DWI-positive group than DWI-negative group. Based on ROC curve analysis (Fig. 1), the comparison of under the curve (AUC) showed superiority of Dawson score (0.610 (95% CI, 0.543–0.678)) and DOT score (0.625 (95% CI, 0.559–0.691)) compared with the ABCD2 score (0.585 (95% CI, 0.517–0.654)) and ABCD3 score (0.609 (95% CI, 0.540–0.678)) (Table 4). ABCD3I score was superior to all other scores (0.83 (95% CI, 0.779–0.881)). The corresponding cutoff values are shown in Table 4. The cutoff values of the ABCD3 score and the DOT score was 5.5 and 0.079, respectively.

**Table 4:** Receiver operating characteristic curve analysis for various scores

Test result variable (s)	Area	*95% CI*	*P* value	Cutoff
ABCD2	0.585	0.517, 0.654	0.019	3.5
ABCD3	0.609	0.540, 0.678	0.003	5.5
ABCD3I	0.830	0.779, 0.881	0.000	6.5
Dawson	0.610	0.543, 0.678	0.002	6.81
DOT	0.625	0.559, 0.691	0.001	0.079

*DWI*, diffusion-weighted imaging; *DOT*, the Diagnosis of TIA score

In addition, we also found that most of the DWI lesions were located in the periventricular area, basal ganglia, and cortical and subcortical regions. Most lesions were diffused punctiform lesions or lacunar infarcts. DWI lesions were more frequent in the anterior circulation.

## Discussion

In our present study, our findings showed that DWI lesions were detected in 29.5% of Chinese patients with transient neurological symptoms. The clinical characteristic associated with DWI lesions was dysphasia. The ABCD2, ABCD3, ABCD3I, Dawson score, and DOT score were significantly higher in the DWI-positive group. The ABCD3I score was superior to all other scores, and the AUC of ABCD3I and DOT score were 0.83 and 0.63, respectively.

The frequency of positive DWI findings was different, which varied from 9 to 67% in patients with transient neurological symptoms in prior studies [12]. In young patients, about 15% of whom demonstrated acute DWI lesions according to brain MRI [16]. In our study, the frequency of an acute DWI lesion was 29.5%, which was consistent with the above prior studies. In our study, there were no differences in the time from onset to DWI examination between the two groups. A potential cause for heterogeneity of the incidence of positive DWI may be the time from symptom onset to DWI examination. A meta-analysis indicated that there was no evidence that the DWI-positive rate varied with time from symptom onset to DWI examination, but it was found that DWI lesions may disappear within 24 h or be undetectable on hyperacute imaging [12]. The study of Shono K also demonstrated that short latency (less than 2 h) from symptom onset to initial DWI was an independent risk factor associated with false-negative findings on DWI [22]. As a result, a repeated DWI examination is recommended for these patients with transient neurological symptoms. Further research is needed to define the relationship between DWI lesions and the time from symptom onset to examination.

Additionally, our study also found that acute DWI lesions were associated with dysphasia, which was also in agreement with previous investigations [14]. To date, the diagnosis of TIA is mainly based on detailed history-taking, thus, the diagnosis of TIA can be difficult and 50–60% of patients seen in TIA clinics turn out to be nonvascular mimics [18]. This may explain why dysphasia is an important underlying factor to be focused on. Episodes of acute atypical or nonfocal neurological symptoms referred to as transient neurological attack (TNA) are as prevalent as TIA. It was reported that DWI showed acute lesions in 23% of patients clinically diagnosed as TNA by experienced stroke neurologists [23]. This raises questions about the accuracy of the clinical diagnosis of TIA. As a result, patients with dysphasia should be taken seriously.

Our result also showed that ABCD2, ABCD3, ABCD3I, Dawson score, and DOT score were significantly higher in DWI-positive group. Furthermore, DWI-positive group had more severe vascular stenosis and a higher level of IMT. In our study, based on ROC, the comparison of AUC showed the superiority of the Dawson score and DOT score compared with the ABCD2 score and the ABCD3 score. The Dawson score is a clinical scoring system that assists with the diagnosis of TIA [21], which was not designed for retinal and some posterior circulation events. The DOT score is a new clinical diagnostic tool for both brain and retinal TIA [18]. With the use of the Dawson and DOT scores, the diagnosis of TIA could be more accurate. It seems to be useful to predict the acute ischemic lesions on DWI with transient neurological symptoms. In addition, our study showed that the ABCD3I score was superior to all other scores. Therefore, the DOT score and ABCD3I score may play an important role in the diagnosis of TIA and assessing the risk of early stroke after TIA in Chinese patients.

The strengths of our study were shown as follows. Firstly, we investigated the incidence of positive DWI in Chinese patients with transient neurological symptoms and identified the independent predictors including laboratory tests and examinations for this positive DWI. Secondly, we validated the DOT score in the Chinese population. DOT score could have a higher predictability of DWI lesions in patients with transient neurological symptoms. Thirdly, the TIA-related scores including ABCD2, ABCD3, ABCD3I, Dawson score, and DOT score were compared in our study.

Our study also has several limitations. First, the samples were small, and our study population was based on hospital patients in a single-center, and there might have been selection bias. A study with a larger number of patients from multiple centers is needed to confirm the characteristics associated with DWI lesions. Second, besides DWI, it was reported that perfusion-weighted imaging (PWI) is useful in defining whether or not the transient neurological symptoms in DWI-negative TIA are true vascular events. The presence of a focal perfusion abnormality is a strong predictor of new DWI lesions at follow-up in DWI-negative TIA patients [24]. We need a variety of imaging tools to determine potential mechanisms underlying such events.

## Conclusion

Our results showed that acute DWI lesions were detected in 29.5% of patients with transient neurological symptoms. Acute DWI lesions were associated with dysphasia. The Dawson score and DOT score may be useful for the early diagnosis and management of TIA. The characteristics associated with DWI lesions need to be confirmed in further studies.
